# Dietary Tributyrin Supplementation Attenuates Insulin Resistance and Abnormal Lipid Metabolism in Suckling Piglets with Intrauterine Growth Retardation

**DOI:** 10.1371/journal.pone.0136848

**Published:** 2015-08-28

**Authors:** Jintian He, Li Dong, Wen Xu, Kaiwen Bai, Changhui Lu, Yanan Wu, Qiang Huang, Lili Zhang, Tian Wang

**Affiliations:** College of Animal Science and Technology, Nanjing Agricultural University, Nanjing, Jiangsu, People’s Republic of China; University of Catanzaro Magna Graecia, ITALY

## Abstract

Intrauterine growth retardation (IUGR) is associated with insulin resistance and lipid disorder. Tributyrin (TB), a pro-drug of butyrate, can attenuate dysfunctions in body metabolism. In this study, we investigated the effects of TB supplementation on insulin resistance and lipid metabolism in neonatal piglets with IUGR. Eight neonatal piglets with normal birth weight (NBW) and 16 neonatal piglets with IUGR were selected, weaned on the 7^th^ day, and fed basic milk diets (NBW and IUGR groups) or basic milk diets supplemented with 0.1% tributyrin (IT group, IUGR piglets) until day 21 (n = 8). Relative parameters for lipid metabolism and mRNA expression were measured. Piglets with IUGR showed higher (*P* < 0.05) concentrations of insulin in the serum, higher (*P* < 0.05) HOMA-IR and total cholesterol, triglycerides (TG), non-esterified fatty acid (NEFA) in the liver, and lower (*P* < 0.05) enzyme activities (hepatic lipase [HL], lipoprotein lipase [LPL], total lipase [TL]) and concentration of glycogen in the liver than the NBW group. TB supplementation decreased (*P* < 0.05) the concentrations of insulin, HOMA-IR, low-density lipoprotein cholesterol, and high-density lipoprotein cholesterol in the serum, and the concentrations of TG and NEFA in the liver, and increased (*P* < 0.05) enzyme activities (HL, LPL, and TL) and concentration of glycogen in the liver of the IT group. The mRNA expression for insulin signal transduction pathway and hepatic lipogenic pathway (including transcription factors and nuclear factors) was significantly (*P* < 0.05) affected in the liver by IUGR, which was efficiently (*P* < 0.05) attenuated by diets supplemented with TB. TB supplementation has therapeutic potential for attenuating insulin resistance and abnormal lipid metabolism in IUGR piglets by increasing enzyme activities and upregulating mRNA expression, leading to an early improvement in the metabolic efficiency of IUGR piglets.

## Introduction

Intrauterine growth retardation (IUGR) is defined as impaired growth and development of the fetus and/or its organs during gestation [1–4], and it has become one of the most important causes for perinatal morbidity and affects about 7–15% pregnancies worldwide [5, 6]. In addition, natural or environmental factors (e.g., nutritional deficiency, disease, and heat stress) that lead to IUGR have been reported in livestock (including pig, sheep, and cattle) [7]. Notably, the risk of IUGR is higher in piglets than in other animals. IUGR has long-term adverse effects on neonatal survival, postnatal growth, nutrition utilization efficiency, health, and performance. Thus, a study on IUGR has important implications for animal science.

The liver plays a critical role in lipid, glucose, and amino acid metabolism, and abnormal metabolism of nutrients in the liver of fetuses with IUGR has been observed [8]. Previous theories have suggested that IUGR are not only linked closely with insulin resistance[9], lipid dysfunction but also fatty liver and inflammation [10]. The mRNA expression for the hepatic lipogenic pathway (including transcription factors and nuclear factors) and enzymes are affected by IUGR, which may be associated with dysfunction in metabolism through the regulation of genes such as liver X receptor α (*LXRα*), peroxisome proliferator-activated receptor α (*PPARα*), and sterol regulatory element-binding protein-1 (*SREBP-1*). Tributyrin (TB), a triglyceride with 3 butyrate molecules, can act as a short-chain fatty acid [11]. A previous study on weaned piglets showed that diets supplemented with 0.1% TB could protect normal intestinal morphology [12]. Other studies have also shown that diets supplemented with TB protect mice from dyslipidemia without affecting food consumption [13] and rats from lipopolysaccharide-induced liver injury [14]. A previous study on Caco-2 cells showed that butyrate causes a noticeable reduction in secreted triglycerides (TG; 27%) and phospholipids (25%) and suggested potential regulation of circulating lipoprotein concentrations [15]. To the best of our knowledge, the role of TB in insulin resistance and lipid metabolism in suckling piglets has not been clarified, and the effects of TB on insulin resistance and lipid metabolism in both humans and livestock with IUGR have not been reported. In order to test this hypothesis, we chose suckling piglets with a low birth weight (LBW) as the IUGR model for investigating whether TB treatment could result in protection against IUGR-induced insulin resistance and associated lipid dysregulation.

## Materials and Methods

### Milk replacer diets

Experimental diets used for the present study were prepared to be isoenergetic and isonitrogenous by supplementation with 0.1% TB (casein-coated; TB content, 50%) and basic milk replacer powder (Table 1). TB was supplied free by Xin Ao Agricultural Development Company (Xia Men, China).

**Table 1:** Composition and nutrient content of the diets.

Items	Control	TB ^a^
Ingredients (g/kg)		
Whey protein concentrates (34%) CP)	300	300
Milk fat powder (11% CP)	350	350
Whole milk powder	200	200
α-Casein	50	50
Glucose	60	60
Pre-mixed mixture ^b^	40	40
TB	0	1
Nutrient content ^c^		
CP (%)	23.31	23.28
Digestible energy (MJ/kg)	14.88	14.88
Lysine (%)	1.51	1.51
Methionine (%)	0.43	0.43
Threonine (%)	0.89	0.89
Tryptophan (%)	0.25	0.25
Ca (%)	0.85	0.85
Total P (%)	0.70	0.71

CP, crude protein; TB, tributyrin; Ca, calcium; P, phosphorus.

^a^ In the tributyrin (TB) diets, 0.1% TB was added to the control diet. TB (casein-coated; net content of TB, 50%) was supplied for free by the Xin Ao Company in Xia Men, China. The 2 diets were isonitrogenous and isoenergetic.

^b^ Main contents of the pre-mixed mixture (per kg of the pre-mixed mixture): Cu (as CuSO_4_·5H_2_O) 600 mg; Fe (FeSO_4_·7H_2_O) 8400 mg; Mn (MnSO_4_·H_2_O) 315 mg; Se (as Na_2_SeO_3_) 17 mg; Zn (ZnSO_4_·7H_2_O) 12500 mg; vitamin A 55000 IU; vitamin D 5500 IU; vitamin E 400 IU; vitamin K 12.5 mg; biotin 2 mg; choline 15 mg; folacin 7.5 mg; riboflavin 100 mg; thiamin 317.5 mg; vitamin B6 175 mg; and vitamin B12 500 mg.

^c^ Nutrient contents of CP, Ca, and P were the examined values; the contents of other nutrients were calculated values.

### Animals and treatment

All the procedures were approved by the Institutional Animal Care and Use Committee of Nanjing Agricultural University. The experiment was performed in An You Trial Pig Farm (Xuancheng, Anhui Province, China). At the time of parturition (day 114 [SD 1] of gestation), Duroc × (Landrace × Yorkshire) neonatal piglets were selected from 8 sows (Landrace × Yorkshire) with similar weight and parity (3^rd^ or 4^th^). The sows were fed a similar gestating diet, according to the nutrient requirements of the National Research Council (NRC, 2012). On the day of delivery, the birth weight and sex of each newborn piglet were recorded. In each litter, piglets with a body weight of 1.67 (SD 0.13) kg and 0.93 (SD 0.11) kg at birth were defined as NBW and IUGR piglets, according to previous studies [16]. The NBW piglets were assigned to the NBW group, and 2 IUGR piglets from 1 litter were randomly assigned to the IUGR and IT (TB supplementation) groups (n = 8/group, 4 males and 4 females). All piglets were weaned on day 7 and fed with warm milk substitute (100 g of milk in 600 ml of warm water) diets per meal every 3 h by using a bottle. The NBW and IUGR groups were fed with a basic milk diet, and the IT group was fed with a basic milk diet supplemented with 0.1% TB to day 21. All piglets were housed individually in pens with plastic floors at an ambient temperature of 33°C in an environmentally controlled room, and they had free access to water.

### Sample collection

At 21 days of age, 6 piglets (3 males and females) with nearly equal body weights (within groups) were selected from each group (n = 8/group) and were weighed prior to euthanization. All piglets were killed by administering an intramuscular injection of sodium pentobarbital (50 mg/kg of body weight) at 2 h after the last meal. Blood samples were obtained by jugular venipuncture and then centrifuged at 3500 r/min for 10 min at 4°C; the serum was stored at -20°C until further analyses. Fresh liver samples were weighed and immediately collected using ice cubes and then stored at -80°C for further analyses.

### Serum hormone concentrations

Concentrations of insulin and leptin in the serum were determined with radioimmunoassays by using kits from Tianjin Nine Tripods Biomedical Engineering, Inc. (Tianjin, China). HOMA-IR = [fasting glucose (mmol/L)×fasting insulin (μU/ml)] / 22.5; HOMA-IR, homeostasis model of assessment for insulin resistence index.

### Serum biochemical parameters and enzyme activities

Concentrations of total cholesterol (TC) and triglycerides (TG) in the serum and liver were measured as described previously [17, 18]. Concentrations of high-density lipoprotein cholesterol (HDL-C), low-density lipoprotein cholesterol (LDL-C), glycogen and non-esterified fatty acid (NEFA) and activities of hepatic lipase (HL) and lipoprotein lipase (LPL) were determined using colorimetric kits (Nanjing Jiancheng Institute of Bioengineering) with a spectrophotometer, according the manufacturer’s instructions. Total lipase (TL) activity was defined as equal to HL and LPL activities.

### Total RNA isolation and relative quantification of gene expression

Total RNA was isolated from the liver samples stored at -80°C by using TRIzol reagent (Invitrogen, Shanghai, China). Determination of RNA content, mRNA quantification, and real-time polymerase chain reaction (PCR; Applied Biosystems, Foster City, CA) were performed according to previously described methods [19]. The primer sequences for the target and reference genes (*INS*, *IRS1*, *PIK3C3*, *Akt2*, *LXRα*, *PPARα*, *CD36*, *SREBP-1*, *FAS*, ACCβ, *SCD*, *FABP*, and *GAPDH*) used for real-time PCR are listed in Table 2. Briefly, a reaction system of 20 μl was composed of 0.4 μl of forward primer, 0.4 μl of reverse primer, 0.4 μl ROX Reference Dye, 10 μl of SYBR Premix Ex Taq (TaKaRa Biotechnology Co. Ltd., Dalian, China), 6.8 μl of double-distilled water, and 2 μl of complementary DNA. The 2^-△△Ct^ method was used to calculate relative levels of mRNA expression after normalization with those of *GAPDH* as a housekeeping gene [20]. The values for the NBW piglets were used for calibration.

**Table 2:** Primer sequences used for quantitative real-time PCR assays.

Gene	Primers	Sequences (5′-3′)			Size(bp)
INSR	Forward	CACCATTCCGGGTTTCTCCA	Reverse	TAGCCAGTGAAGTGACGCAG	129
IRS1	Forward	GCCACGGGAGAATGGGTTTA	Reverse	GTCGCACACAGTTTCAGCAG	73
PIK3C3	Forward	GCATGTTTCGCCAAGGGATG	Reverse	CTGCTTGTTCTGCCAGGAGT	94
Akt2	Forward	CTGACCTGCTGTCCGCAAAT	Reverse	GACACGCTGTCACCTAGCTT	161
SREBP-1	Forward	GCTACCGCTCCTCCATCAAT	Reverse	CTGCTTGAGCTTCTGGTTGC	146
LXRα	Forward	CCCTCTCTCGCTCAGCTCC	Reverse	GGAGCCCTGGACATTACCAA	150
PPARα	Forward	CTGGCCACATCCATCCAACA	Reverse	ATAACGGGCTTTCCAGGTCG	89
FAT	Forward	TAGGAATCCCACTGCCTCAC	Reverse	TGCTTCAAGTGCTGGGTCA	113
FAS	Forward	ATCAACCCTGCTTCCCTTCG	Reverse	ATCATGCTGTAGCCCACGAG	116
ACCβ	Forward	CCCAACTCTCGCTCGTTTCA	Reverse	AACCCAACCATGGAGAGGAG	80
SCD	Forward	TGCTGATCCCCACAATTCCC	Reverse	CTTTGACGGCTGGGTGTTTG	83
FABP	Forward	GAGTAGCCTCATTGCCACCAT	Reverse	TGCACGATTTCCGATGTCCC	147
GAPDH	Forward	CATTGCCCTCAACGACCACT	Reverse	ATGAGGTCCACCACCCTGTT	84

INSR, insulin receptor; IRS1, insulin receptor substrate 1; PIK3C3, phosphatidylinositol 3-kinase, catalytic subunit type 3; Akt2, alanine aminotransferase assav 2; SREBP-1, sterol regulatory element-binding protein-1; LXRα, liver-x receptor α; PPARα, peroxisome proliferator-activated receptor α; FAT/CD36, fatty acid translocate; FAS, fatty acid synthesis; ACCβ, acetyl-CoA carboxylase β; SCD, stearoyl-CoA desaturase; FABP, fatty acid-binding protein; GAPDH, glyceraldehyde-3-phosphate dehydrogenase.

### Statistical analysis

One-way analysis of variance was used to determine the main effects of IUGR and the diets, followed by multiple pairwise comparisons (Duncan method, used for multi-group comparisons) by using SPSS 17.0. (SPSS, Inc., Chicago, IL). *P* values lower than 0.05 were considered as statistically significant, and those lower than 0.01 were considered as very significant. Data are presented as mean ± standard error values.

## Results

### Serum hormone concentrations

IUGR caused a significant increase (*P* < 0.05) in the concentration of insulin in the serum and HOMA-IR. TB decreased (*P* < 0.05) the concentration of insulin in the serum and HOMA-IR of IUGR piglets. HOMA-IR of the IT group was lower (*P* < 0.05) than that of the NBW group. There were no significant differences (*P* > 0.05) in leptin concentration in the serum among the 3 groups (Table 3). Low concentration of glucose was observed in the serum of piglets with IUGR (P > 0.05). TB increased the concentration of glucose in the serum of piglets with IUGR (P > 0.05) (Data not shown).

**Table 3:** Effect of tributyrin (TB) on the concentrations of insulin and leptin in the serum of piglets with intrauterine growth retardation (day 21).

Items	NBW Group	IUGR Group	IT Group
Insulin (pmol/l)	249.39 ± 14.391^b^	380.072 ± 36.384^a^	276.953 ± 8.257^b^
Leptin (nmol/l)	0.102 ± 0.011	0.091 ± 0.017	0.080 ± 0.004
HOMA-IR	5.252±0.303^b^	6.609±0.633^a^	3.623±0.108^c^

Data are expressed as mean ± SEM, n = 6; SEM, standard error of mean.

^a,b,c^ Mean values within a line with different superscript letters were significantly different (*P* < 0.05). NBW, piglets with a normal birth weight that were fed with basic milk diets; IUGR: piglets with intrauterine growth retardation that were fed with basic milk diets; IT, piglets with intrauterine growth retardation that were fed with diets supplemented with 0.1% TB. HOMA-IR = [fasting glucose (mmol/L)×fasting insulin (μU/ml)] / 22.5; HOMA-IR, homeostasis model of assessment for insulin resistence index.

### Relative serum and liver parameters

IUGR caused a significant decrease (*P* < 0.05) in the concentration of glycogen in the liver. TB increased (*P* < 0.05) the concentration of glycogen in the liver of IUGR piglets. Compared to the NBW group, the IUGR group showed increased concentrations of TC and TG (*P* < 0.05). TB increased (*P* < 0.05) the concentration of TG in the serum and decreased (*P* < 0.05) the concentration of LDL-C and HDL-C in the serum and TG in the liver, when compared with the IUGR group. No significant (*P* > 0.05) differences were observed between the NBW and IT groups, except the IT group showed a higher concentration of TC in the liver than the NBW group (*P* < 0.05; Table 4).

**Table 4:** Effects of tributyrin (TB) on concentrations of total cholesterol (TC), triglycerides (TG), low-density lipoprotein cholesterol (LDL-C), high-density lipoprotein cholesterol (HDL-C) in the serum and glycogen, TC, TG, non-esterified fatty acid (NEFA) in the liver of piglets with intrauterine growth retardation (IUGR) (day 21).

Items		NBW Group	IUGR Group	IT Group
**Serum**				
	TC (mmol/L)	2.054 ± 0.044	2.089 ± 0.061	1.993 ± 0.122
	TG (mmol/L)	0.423 ± 0.037^a^ ^b^	0.340 ± 0.024^b^	0.532 ± 0.047^a^
	LDL-C (mmol/L)	0.337 ± 0.025^a^ ^b^	0.417 ± 0.100^a^	0.203 ± 0.043^b^
	HDL-C (mmol/L)	0.723 ± 0.019^a^ ^b^	0.788 ± 0.036^a^	0.645 ± 0.025^b^
**Liver**				
	Glycogen (mg/g protein)	2.324 ±0.293^a^ ^b^	1.856 ±0.236^b^	3.132 ±0.341^a^
	TC (mmol/g protein)	0.023 ± 0.001^b^	0.027 ± 0.001^a^	0.029 ± 0.001^a^
	TG (mmol/g protein)	2.015 ± 0.238^b^	5.128 ± 0.676^a^	2.000 ± 0.166^b^
	NEFA(μmol/g protein)	326.951 ± 43.952^c^	735.965 ± 43.387^a^	558.567 ± 12.074^b^

TC, total cholesterol; TG, triglycerides; LDL-C, low-density lipoprotein cholesterol; HDL-C, high-density lipoprotein cholesterol; NEFA, non-esterified fatty acid. Data are expressed as mean ± SEM, n = 6. SEM, standard error of mean.

^a,b,c^ Mean values within a line with different superscript letters were significantly different (*P* < 0.05). NBW, piglets with normal birth weight that were fed with basic milk diets; IUGR: piglets with intrauterine growth retardation that were fed with basic milk diets; IT, piglets with intrauterine growth retardation that were fed with diets supplemented with 0.1% tributyrin.

### Hepatic lipase, lipoprotein lipase, and total lipase activities

Compared to the NBW group, the IUGR group showed decreased (*P* < 0.05) activities of HL, LPL, and TL in terms of per milligram protein in the liver. TB increased (*P* < 0.05) HL, LPL and TL activities in the liver compared with that of IUGR group. There was no significant differences (*P* > 0.05) of HL, LPL and TL between IT group and NBW group (Table 5).

**Table 5:** Effect of tributyrin on the activities of hepatic lipase (HL), lipoprotein lipase (LPL), and total lipase (TL) in the liver of piglets with intrauterine growth retardation (IUGR) (day 21).

Items	NBW Group	IUGR Group	IT Group
HL (U/mg protein)	0.579 ± 0.052^a^	0.222 ± 0.042^b^	0.678 ±0.068^a^
LPL (U/mg protein)	0.362 ± 0.024^a^	0.125 ± 0.043^b^	0.262 ± 0.036^a^
TL (U/mg protein)	1.095 ± 0.102^a^	0.402 ± 0.084^c^	0.776 ± 0.066^b^

HL, hepatic lipase; LPL, lipoprotein lipase; TL, total lipase, equal to hepatic lipase and lipoprotein lipase activities; Data are expressed as mean ± SEM, n = 6. SEM, standard error of mean.

^a,b,c^ Mean values within a line with different superscript letters were significantly different (*P* < 0.05). NBW, piglets with normal birth weight that were fed with basic milk diets; IUGR: piglets with intrauterine growth retardation that were fed with basic milk diets; IT, piglets with intrauterine growth retardation that were fed with diets supplemented with 0.1% tributyrin.

### mRNA expression of lipid-related genes in the liver of piglets with intrauterine growth retardation (day 21)

**Fig 1:**
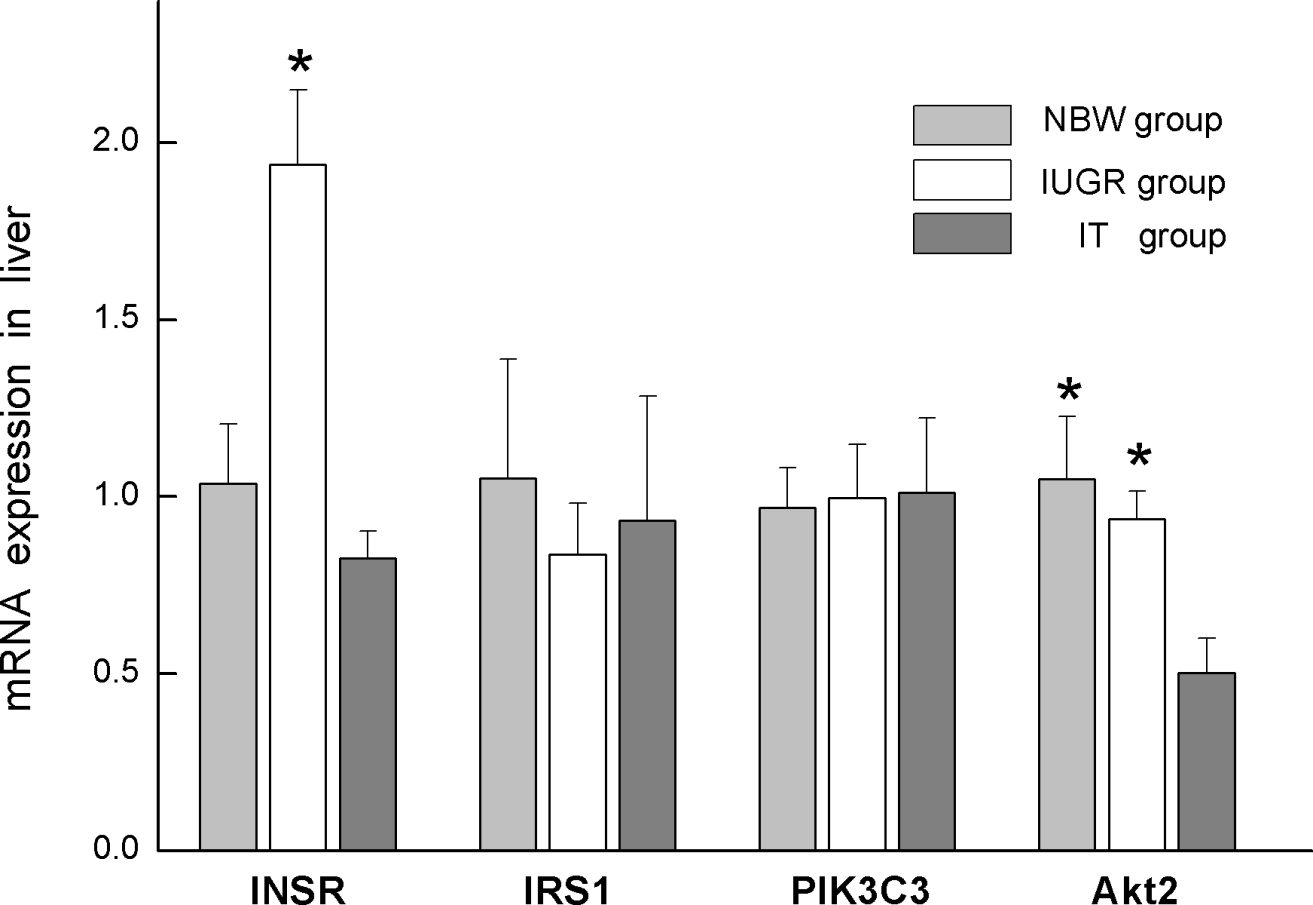
mRNA expression for INSR, IRS1, PIK3C3, and Akt2 was normalized using GAPDH as the control. Data are expressed as mean ± SEM, n = 6. SEM, standard error of mean. * indicates a significant difference from control group (*P* < 0.05). NBW, piglets with normal birth weight that were fed with basic milk diets; IUGR: piglets with intrauterine growth retardation that were fed with basic milk diets; IT, piglets with intrauterine growth retardation that were fed with diets supplemented with 0.1% tributyrin.

Compared to the NBW group, the IUGR group showed marked (*P* < 0.05) upregulation of mRNA expression for INSR. There was no significant difference (*P* > 0.05) of Akt mRNA expression between NBW and IUGR group. The mRNA expression for INSR and Akt2 in the IT group was downregulated significantly (*P* < 0.05) in comparison with that in the IUGR group, but INSR was not significantly different (*P* > 0.05) from the NBW group. There was no significant differences of IRS1 and PIK3C3 mRNA expression among the 3 groups (Fig 1).

**Fig 2:**
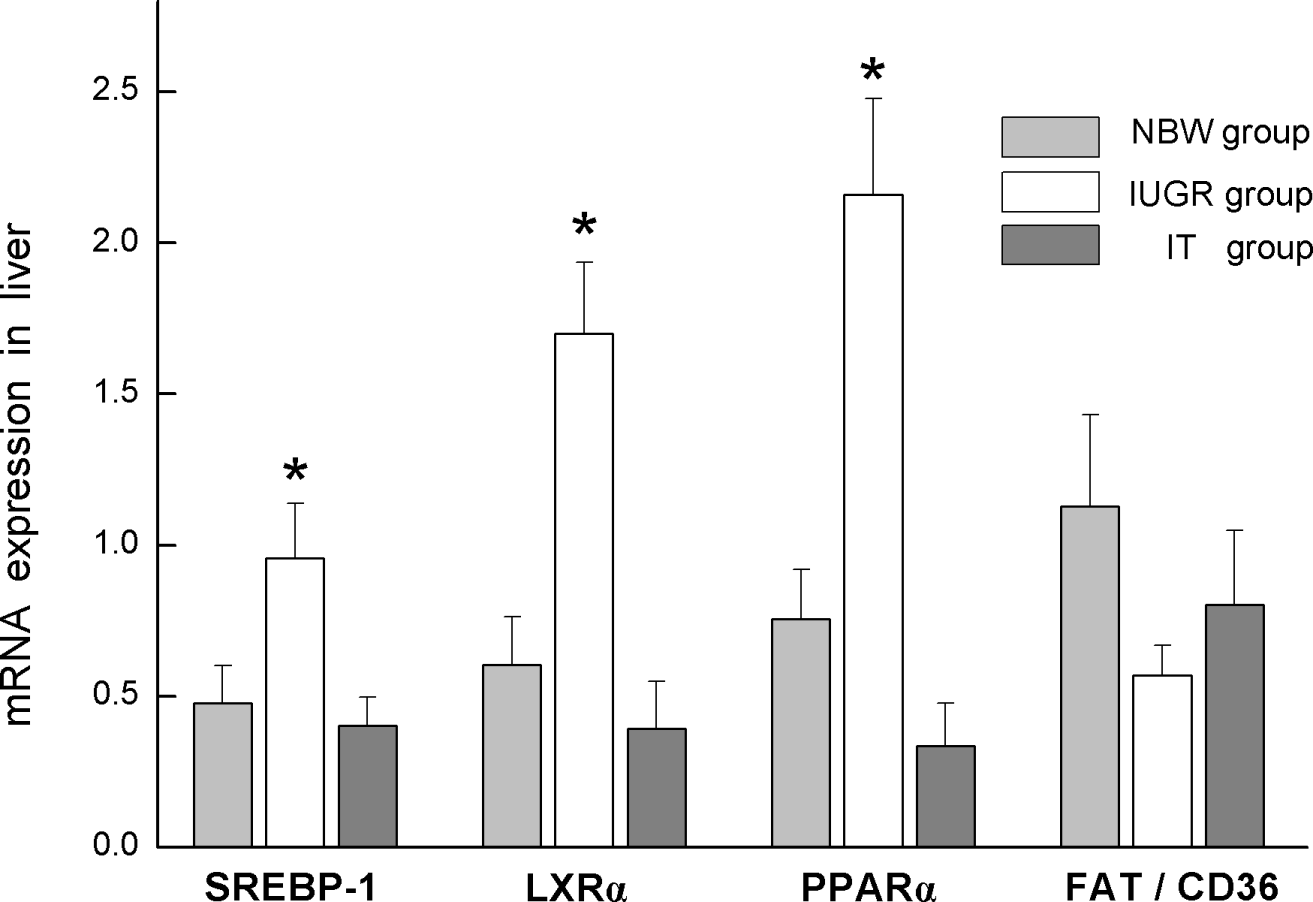
mRNA expression for SREBP-1, LXRα, PPARα, and FAT/CD36 was normalized using GAPDH as the control. Data are expressed as mean ± SEM, n = 6. SEM, standard error of mean. * indicates a significant difference from control group (*P* < 0.05). NBW, piglets with normal birth weight that were fed with basic milk diets; IUGR: piglets with intrauterine growth retardation that were fed with basic milk diets; IT, piglets with intrauterine growth retardation that were fed with diets supplemented with 0.1% tributyrin.

Compared to the NBW group, the IUGR group showed marked (*P* < 0.05) upregulation of mRNA expression for SREBP-1, LXRα, and PPARα. The mRNA expression for SREBP-1, LXRα, and PPARα in the IT group was downregulated significantly (*P* < 0.05) in comparison with that in the IUGR group and was not significantly different (*P* > 0.05) from the NBW group. There was no significant difference of FAT/CD36 mRNA expression among the 3 groups (*P* > 0.05; Fig 2).

**Fig 3:**
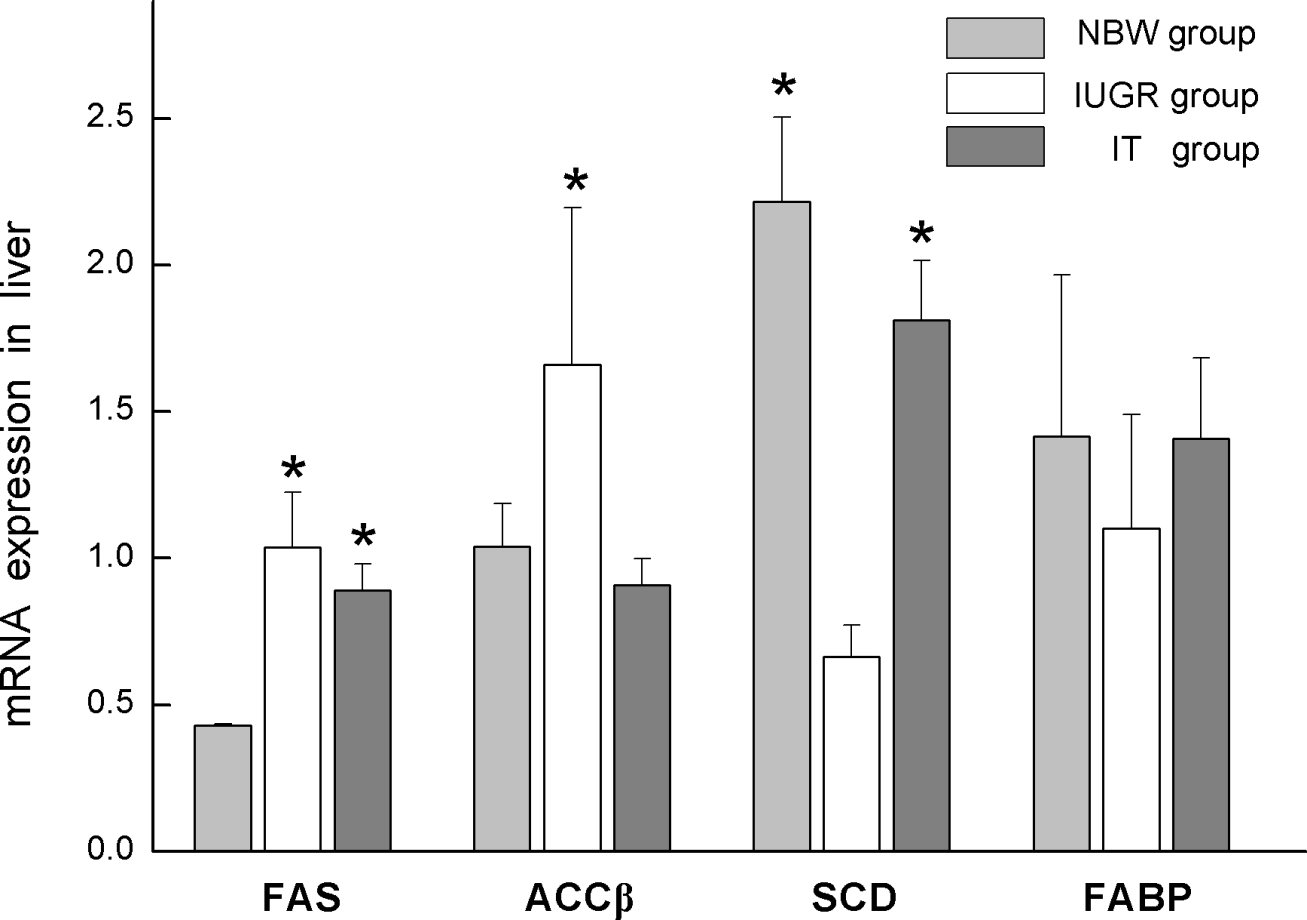
mRNA expression of FAS, ACCβ, SCD, and FABP was normalized using GAPDH the control. Data are expressed as mean ± SEM, n = 6. SEM, standard error of mean. * indicates a significant difference from control group (*P* < 0.05). NBW, piglets with normal birth weight that were fed with basic milk diets; IUGR: piglets with intrauterine growth retardation that were fed with basic milk diets; IT, piglets with intrauterine growth retardation that were fed with diets supplemented with 0.1% tributyrin.

Notably, mRNA expression for FAS and ACCβ was increased in the IUGR group (*P* < 0.05). TB supplementation decreased the mRNA expression for ACCβ (*P* < 0.05) and FAS (*P* > 0.05) in IUGR piglets. The mRNA expression for SCD was decreased (*P* < 0.05) in the IUGR group, but it was increased obviously (*P* < 0.05) in the IT group. However, IUGR and TB diets did not significantly affect the mRNA expression for FABP (*P* > 0.05; Fig 3).

## Discussion

The development of IUGR neonates is impaired, and they are born with a lower birth weight [7, 21] and associated with several metabolic syndromes in adulthood [22]. IUGR also induces insulin resistance, liver injuries and impairs liver metabolism early in pigs [8, 9]. Abnormal metabolism of nutrients, such as abnormal fatty acid synthesis and lipid oxidation occurs in the liver of IUGR fetuses [4, 7, 23]. Butyric acid is regarded as one of the most efficient short-chain fatty acids, with relevant effects on the development of obesity [24]. Previous studies have demonstrated that butyric acid efficiently improves the development of animals and protects them from injury [25, 26]. TB has already been confirmed to increase average day gain in weaned pigs [27], and it improves intestinal digestive and barrier functions in IUGR piglets during the suckling period [28]. In the present study, we investigated the potential regulatory mechanism of IUGR treatment with 0.1% TB.

Epidemiological studies have shown that IUGR is strongly associated with the onset of insulin resistance [29]. Insulin, secreted by pancreatic β-cells, efficiently decreases the concentration of blood glucose and promotes fat synthesis. Insulin resistance in white adipose tissue results in increased fatty acid flux to the liver, with subsequent ectopic fat deposition in hepatocytes [30]. Insulin resistance, which can be regarded as the damage of insulin signal transduction, occurs when the normal concentrations of hormone in the circulating are insufficient to regulate metabolic pathways. In the present study, IUGR increased insulin levels in the serum and HOMA-IR, which were used as markers of insulin resistance. The low glucose levels in the serum of IUGR piglets corresponded to the result of high insulin levels. During early time of life, the metabolism of nutritions in IUGR neonatus are not mature (including glucose, proteins, lipids) which results in body accelerating the process of glycolysis for providing energy and slowing down glycogen synthesis. The decreased concentration of glycogen in the liver of IUGR piglets was observed in the present study. The datas may be helpful in proving the complicated metabolism in IUGR during early time. Insulin resistance is usually related to obesity [31] and is found along with fatty liver in patients [32]. Our findings were consistent with those of Eleni *et al*. [33]. The results indicated that IUGR caused glycometabolism disorder [34], which is related to insulin resistance. Other studies have reported an increased concentration of insulin on day 90 of gestation [35] and a decreased concentration of glucose in the serum of IUGR piglets [36]. Previously researches demonstrated that children exposed to IUGR [37] and rats with IUGR [9] have increased insulin resistance. Insulin resistance was closely correlated with the regulation of mRNA expression. Insulin signal transduction pathway shows that insulin binding to the insulin receptor (INSR) mediates the metabolic functions of insulin in liver. The combination directly results in the phosphorylation of tyrosine on insulin receptor substrates (IRS1, IRS2), which provokes phosphatidylinostol 3-kinase (PI3K) and the followed phosphorylation of Akt’s activation [38]. In the present study, IUGR upregulated the mRNA expression of INSR in liver, and had no obvious alterations of IRS1, PIK3C3 and Akt2. These data may be in favor of that high concentrations of insulin in IUGR bingding to the INSR resulted in the subsequent phosphorylation of Akt in the liver and led to the decreased glucose in the serum. However, supplementation with TB protected IUGR piglets from hyperinsulinism and maintained normal metabolism of glycose in the liver of IUGR piglets, and the mRNA expression of INSR and Akt2 was downregulated which corresponded to the decreased concentration of insulin. Insulin sensitivity was evaluated by peripheral glucose uptake and free fatty acid concentrations. These results may indicated that hyperinsulinism in IUGR was related to insulin resistance and the disorder of glycometabolism and lipid catabolism in liver. The specific mechanisms underlying insulin resistance induced by IUGR and attenuated by TB were not clear. Leptin is produced mainly by the adipose tissue in the mammary gland, which is in fact an additional source of leptin during the early phase of lactation, and transported from the mother to newborns. An interesting hypothesis suggests that altered development during fetal and neonatal life could lead to inefficient use of leptin, called “leptin resistance” [39]. In the present study, a decreased concentration of leptin was observed in the serum of IUGR piglets. Lower serum leptin concentrations in IUGR newborns [40] indicate that leptin may be positively correlated with body weight and body mass index. High insulin and low leptin levels in IUGR suckling piglets may indicate dysfunction due to IUGR. These results indicate that TB was quite helpful in attenuating insulin resistance in IUGR suckling piglets, but not the leptin concentration.

Hepatic lipid accumulation results from an imbalance between lipid availability and lipid disposal and eventually triggers lipoperoxidative stress and hepatic injury [41]. LDL is a major transport protein for cholesterol in human plasma [42]. Triacylglycerols are the most common non-toxic form of fatty acids. Triacylglycerols accumulate in hepatocytes unless NEFAs are oxidized or triacylglycerols are exported as constituents of very low-density lipoproteins. In the present study, the concentration of serum TG had a trend to be reduced, and the concentrations of TC, LDL-C, and HDL-C in the serum of IUGR piglets were likely to be increased. Lin *et al*. [35]observed that IUGR fetuses have lower concentrations of TG and higher concentrations of TC, LDL-C, and HDL-C than normal fetuses on day 90 and day 110 of gestation. IUGR piglets showed increased lipogenesis [36], possibly associated with gene expression. Another study also showed that the concentration of TG in the blood on day 28 in IUGR piglets was lower than that in NBW piglets [43]. TC and TG regulated the key steps in intra-fetal synthesis and lipid catabolism [44], and were 2 key markers that reflected circulation blood fat in the body. When high concentrations of NEFA are present, the liver can actively synthesize triacylglycerol. We also observed higher concentrations of TC and TG in the liver of IUGR piglets. The concentration of NEFA in the liver of IUGR piglets was increased, which may result in an increased concentration of TG in the liver. Therefore, transport and metabolism of lipids might have been affected by IUGR in the present study; this finding was also reported by previous studies [45, 46]. However, after TB treatment, lipogenesis could be efficiently attenuated to a normal state, with no obvious differences with respect to NBW piglets. TB treatment is associated with reduced phosphorylated JUN-NH2 terminal kinase content and a partial reversion of hepatic steatosis, and it reduces fat accumulation induced by high-fat feeding [13] or a diet supplemented with butyrate [47].

Importantly, activities of HL, LPL, and TL were decreased by IUGR in the present study. LPL catalyzes the hydrolysis of the triacylglycerol component in circulating chylomicrons and very low-density lipoproteins, thus supplying NEFA for tissue utilization [48]. HL plays a critical role in hydrolyzing triglycerides and phospholipids present in circulating blood. Liu *et al*. [8] observed that IUGR fetuses had lower LPL activity than normal fetuses. These data indicated low enzyme activities in the liver of IUGR fetuses, which could directly result in high concentrations of NEFA and TG in the liver. After TB treatment, HL, LPL, and TL activities were increased in the liver in the IT group, which caused a decrease in the concentrations of NEFA and TG.

Liver plays a crucial role in the regulation of gene expression for key transcription factors [49], including PPARα, SREBP-1C, and LXRα. Regulation of lipogenic gene expression by insulin and fatty acids is largely mediated by transcription factors (such as SREBPs) [50] and in proportion by nuclear receptors (such as LXRs) [51]. Overall, triacylglycerol synthesis is controlled by transcription factors and nuclear receptors [52] and their ligands. PPARα activation would suppress the LXR-SREBP-1c pathway through reduction of LXR/RXRα formation [53]. LXRα would regulate hepatic peroxisomal fatty acid β-oxidation, and this process might be a counter regulatory mechanism for responding to extreme situations such as hypertriglyceridemia and liver steatosis [54]. In the present study, fatty acid translocase (FAT/CD36) mRNA expression was downregulated, and LXRα and PPARα mRNA expressions were upregulated in the liver in IUGR piglets. IUGR was also found to induce the downregulation of SCD and FABP mRNA expression and the upregulation of SREBP-1, FAS, and ACCβ mRNA expression in the liver. These data may be helpful in proving that a high concentration of insulin results in abnormal levels of lipid metabolism via gene expression. By treatment of IUGR with TB, mRNA expressions of these genes could be regulated to normal levels. However, mRNA expression of FAS in the IT group was higher than that in the NBW group, but lower than that in the IUGR group. NEFA enters the cell via transporters (fatty acid transport) or FAT/CD36 or diffusion [49]. Yu *et al*. [55] studied obese mice induced by a high fat diet and showed that treatment with diacylglycerol acyltransferase 2 resulted in an obvious reduction in hepatic triglyceride content and plasma triglyceride-rich lipoprotein via downregulation of the hepatic lipogenic pathway (including SCD1 and ACC) and upregulation of free fatty acid oxidation in hepatocytes. Similarly, TB treatment in the present study resulted in downregulation of gene expression (including those needed for the hepatic lipogenic pathway) in the liver of IUGR piglets and a decrease in NEFA and TG levels in the liver. These observations suggest the abnormal status of lipid metabolism in the IUGR piglets. TB was necessary in facilitating the recovery of IUGR fetuses by promoting lipid decomposition in liver, maintaining stability of glucose metabolism and gene expression.

## Conclusions

In conclusion, our findings clearly demonstrated that piglets with IUGR can lead to the hyperinsulinism and high HOMA-IR, which were closely linked to the insulin resistance. Disorder of glycometabolism and lipid catabolism in the liver of IUGR piglets, and with alterations for gene expression were observed in this study. Importantly, the most remarkable finding of this study was that diet supplementation with 0.1% TB could efficiently attenuate insulin resistance and abnormal levels of lipid metabolism caused by IUGR through regulation of mRNA expression and increasing enzyme activity, which could be helpful in finding a new strategy for early attenuation of insulin resistance and metabolic abnormality caused by IUGR in humans.
